# Bayesian Action&Perception: Representing the World in the Brain

**DOI:** 10.3389/fnins.2014.00341

**Published:** 2014-10-30

**Authors:** Gerald E. Loeb, Jeremy A. Fishel

**Affiliations:** ^1^SynTouch LLCLos Angeles, CA, USA; ^2^Department of Biomedical Engineering, University of Southern CaliforniaLos Angeles, CA, USA

**Keywords:** Bayesian exploration, touch, tactile sensing, perception, somatosensory, affordance

## Abstract

Theories of perception seek to explain how sensory data are processed to identify previously experienced objects, but they usually do not consider the decisions and effort that goes into acquiring the sensory data. Identification of objects according to their tactile properties requires active exploratory movements. The sensory data thereby obtained depend on the details of those movements, which human subjects change rapidly and seemingly capriciously. Bayesian Exploration is an algorithm that uses prior experience to decide which next exploratory movement should provide the most useful data to disambiguate the most likely possibilities. In previous studies, a simple robot equipped with a biomimetic tactile sensor and operated according to Bayesian Exploration performed in a manner similar to and actually better than humans on a texture identification task. Expanding on this, “Bayesian Action&Perception” refers to the construction and querying of an associative memory of previously experienced entities containing both sensory data and the motor programs that elicited them. We hypothesize that this memory can be queried (i) to identify useful next exploratory movements during identification of an unknown entity (“action for perception”) or (ii) to characterize whether an unknown entity is fit for purpose (“perception for action”) or (iii) to recall what actions might be feasible for a known entity (Gibsonian affordance). The biomimetic design of this mechatronic system may provide insights into the neuronal basis of biological action and perception.

## Can robots perceive in the same manner as humans?

How can we reconcile the apparently chaotic behavior of humans exploring their environment with their extremely effective judgment? The brains of higher primates are extraordinarily good at interpreting, learning, and recognizing complex situations and formulating appropriate responses to them, yet the details of their behaviors while doing so are maddeningly noisy to the experimental observer and inconsistent with the deterministic behavior expected of optimally engineered systems. Humans observing a visual scene cause their eyes to dart in sequences of saccades that seem capricious and are often repetitive (Yarbus and Riggs, 1967). Humans trying to identify an object by touch select specific movements according to the relevant properties (Jones and Lederman, 2006) but the details tend to be inconsistent and sometimes repetitive, frequently changing posture, velocity, and force of the fingers (Morley et al., 1983; Smith et al., 2002, 2009). These exploratory behaviors are neither replicable nor “machinelike” in their observable details. Nevertheless, the improvements in elegance, speed, and accuracy of object identification as children develop (Morrongiello et al., 1994) suggests that exploratory movements are chosen carefully on the basis of experience.

The internal details of neural activity that must ultimately be the cause of exploratory behaviors are also not machinelike or consistent. Even when the motor behavior is tightly constrained to be repetitive, the activity of the individual neurons in the cerebral cortex varies wildly from trial to trial (Churchland et al., 2006; Churchland and Shenoy, 2007). This neural activity is not random, as can be seen by averaging data from many trials and extracting correlations with specific features of the behavior. It is tempting to ascribe the inter-trial variability to “computational noise” and to assume that it is overcome during individual trials by averaging together the activity of millions of noisy neurons. Nevertheless, an engineer would wonder how such a badly designed machine manages to perform so remarkably well.

We encountered this paradox when trying to figure out how to use a **biomimetic** tactile sensor (BioTac® from SynTouch LLC, Los Angeles) to enable robots to perform haptic identification (Fishel and Loeb, 2012a). The sensor has mechanical properties and sensing modalities that are similar to the human fingertip (Fishel et al., 2008; Wettels et al., 2008; Fishel and Loeb, 2012b; Wettels et al., 2013), making it an ideal candidate for investigating artificial humanlike tactile characterization of textures. We assumed that we could program the robot to make the same exploratory movements that have been observed in humans exploring textures (Jones and Lederman, 2006) and then subject the sensory signals to standard signal processing and classification schemes to associate them with distinctive properties of the object's texture. Humans actively exploring surfaces use a wide variety of rapidly changing forces and velocities, but they vary inexplicably between subjects and between presentations of a single texture to a single subject; nevertheless, subjects discriminate surfaces accurately and fairly rapidly (Morley et al., 1983; Smith et al., 2002, 2009). We initially considered that the movements observed in humans might simply be noisy and their details irrelevant, so we tried to compute features (such as spectral patterns in skin vibrations) that might be constant and distinctive for textures over a wide range of movement parameters. Through experimentation it became clear that those extracted features actually did depend strongly on the contact force and velocity of the exploratory movements. Furthermore, there was no predictable relationship across objects; e.g., increased sliding velocity might increase a particular sensory signal for one texture but decrease it for another. Interpreting the tactile data required judicious selection and consideration of these exploratory parameters.

KEY CONCEPT 1. Biomimetic Mechatronic systems whose designs and functions are enabled by features and principles of operation found in biological systems.

**Figure 1:**
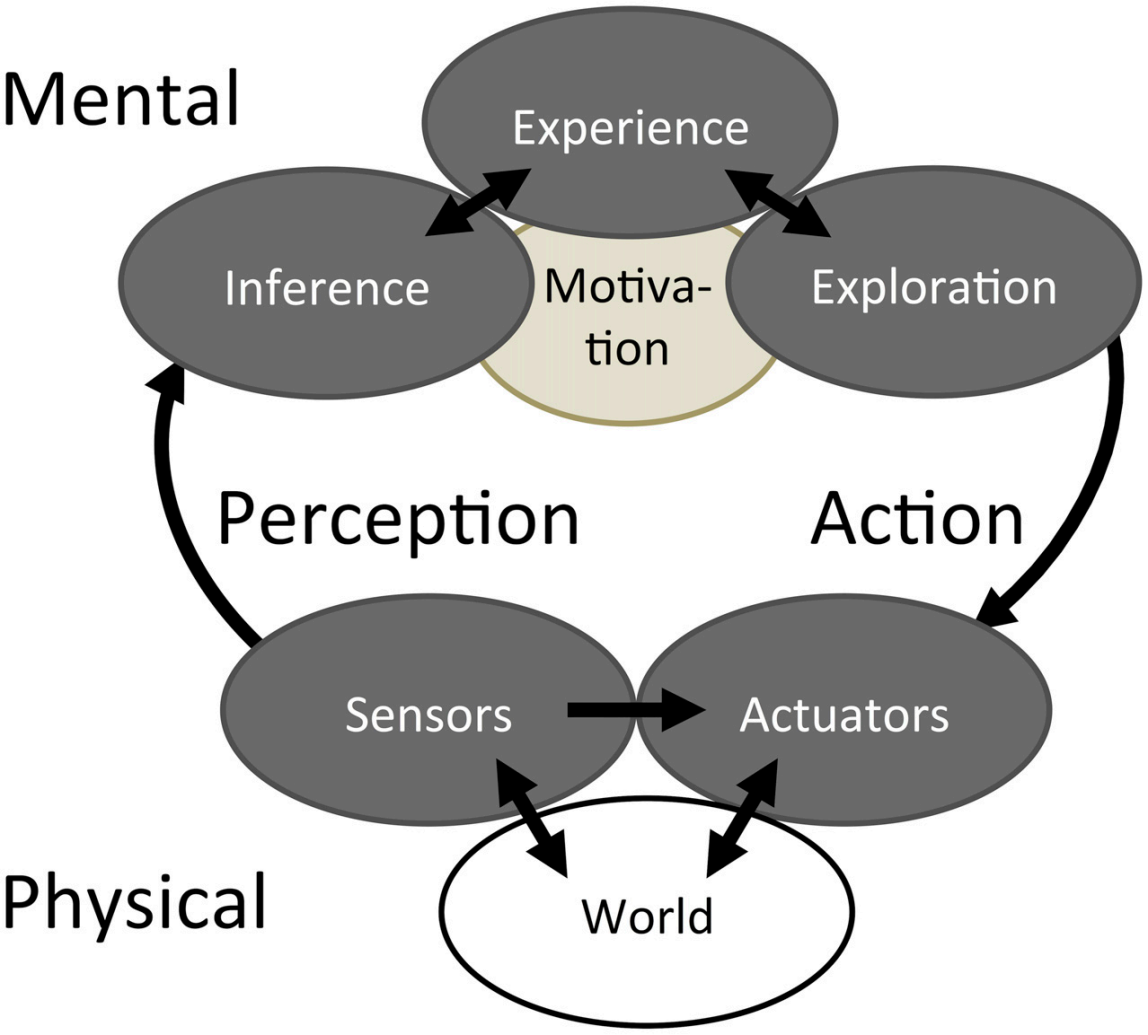
**Bayesian Action&Perception**. A haptic system interacts with the world through a mechanical Interface that provides access to both Sensors and Actuators. These Physical components are connected to Mental functions through the activities of Perception and Action. The Mental functions consist of Bayesian Inference and Bayesian Exploration, both capable of accessing and adding to an associative memory of Experience according to the Motivation to achieve certain goals.

This review considers what high level strategies and internal representations might account for complete behaviors that include both action and perception. The functional steps are illustrated schematically for haptic behaviors in Figure 1 and discussed later in terms of their possible implementation by the brain. This review attempts to unify the concepts of perception and action by drawing parallels between the visual system, which is usually considered from the perceptual perspective, and the haptic system, which is usually considered from the action perspective.

## Decision-making and iterative exploration

The exploratory movements that humans make are variable but not because they are chosen randomly or degraded by motor noise. The sequencing and details of exploratory movements seem to be important, yet not preconceived. **Bayesian inference** lends itself to iterative problem-solving in that each new piece of observed data can be used to update the probabilities of competing hypotheses (Bayes and Price, 1763). It starts with a distribution of “prior probabilities” each describing the initial probability that a particular hypothesis is true (e.g., the probability that the object about to be explored is made of wood, metal, plastic, etc.). After new information is received, it is possible to compute a new distribution of “posterior probabilities” based on the likelihood that each hypothesis, if true, would have resulted in this information (i.e., if the object were made of wood, metal, or plastic would it feel like what was just perceived?).

KEY CONCEPT 2. Bayesian inference A statistical process for computing the probability (*P*) that a hypothesis (*H*) is true given new evidence (*E*) according to Bayes' rule:

$$P(H|E)=\frac{P(E|H)\cdot P(H)}{P(E)}$$

where *P(E|H)* is the probability of observing *E* given that *H* is true; *P(H)* is the prior probability that *H* is true before observing *E*; and *P(E)* is the probability of observing *E* with consideration to all possible hypotheses that could be true.

**Figure 2:**
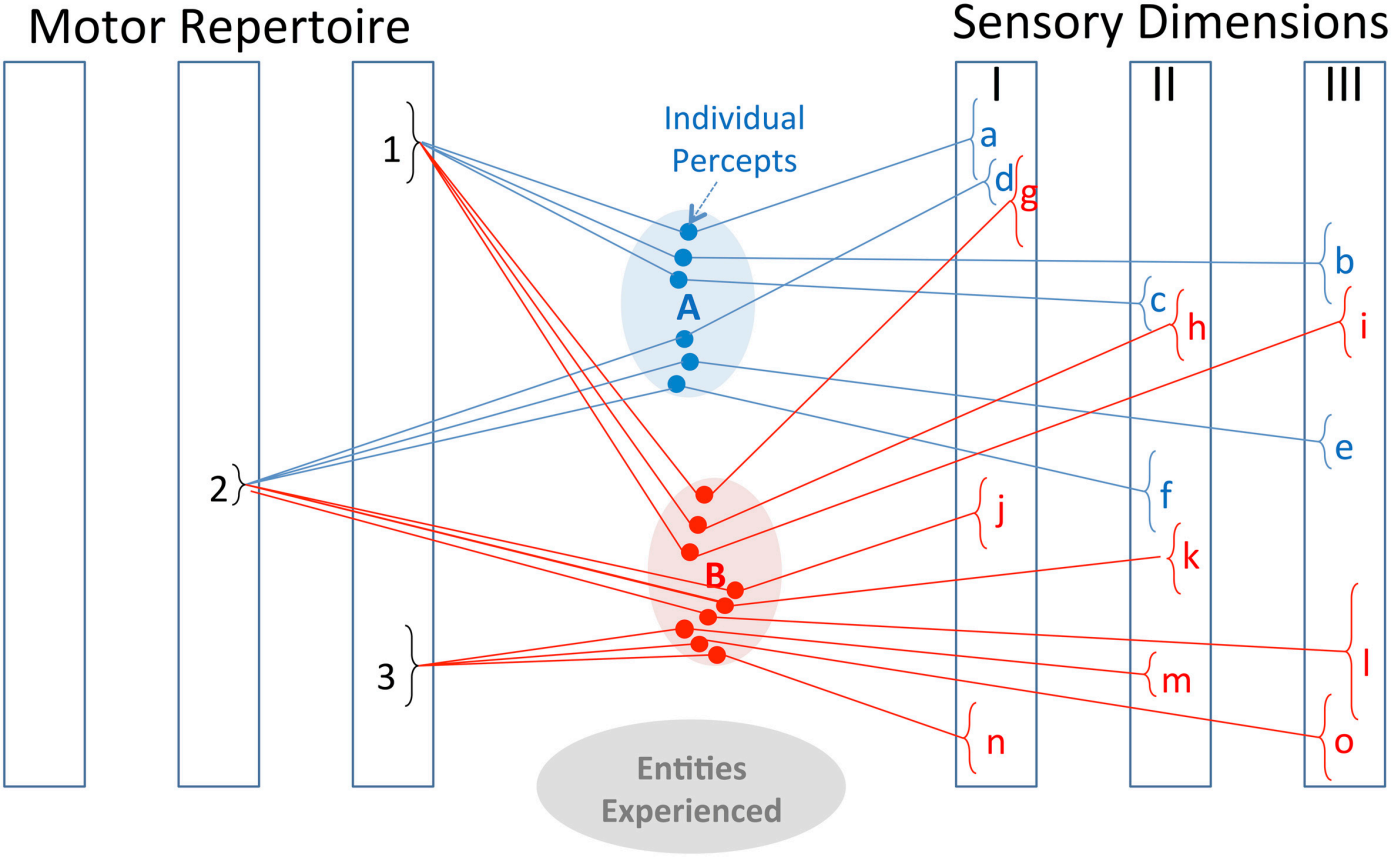
**Bayesian representation**. The internal representations of entities A and B consist of the previously experienced associations of various motor behaviors with various sensory feedback. The motor repertoire consists of discrete types of movements with continuously adjustable parameters; the sensory dimensions represent processed sensations with continuous ranges of values. Discrimination and identification of entities depends on finding motor-sensory associations that distinguish among the alternatives that are currently most probable. When explored by motor strategy 1, entities A and B result in overlapping sensory percepts a, b, c and g, h, i, respectively. When explored by motor strategy 2, the resulting percepts are non-overlapping for two of the three sensory dimensions (d, e vs. j, l), so this is the better exploratory strategy to pursue. Previous experience has also associated entity B with an affordance consisting of the tendency to produce sensory percepts n, m, o when handled according to motor strategy 3. Once a new object has been identified as entity B (or sufficiently close to it), it is immediately obvious that a behavioral result associated with sensory percepts n, m, and o can be obtained by generating motor strategy 3.

**Figure 3:**
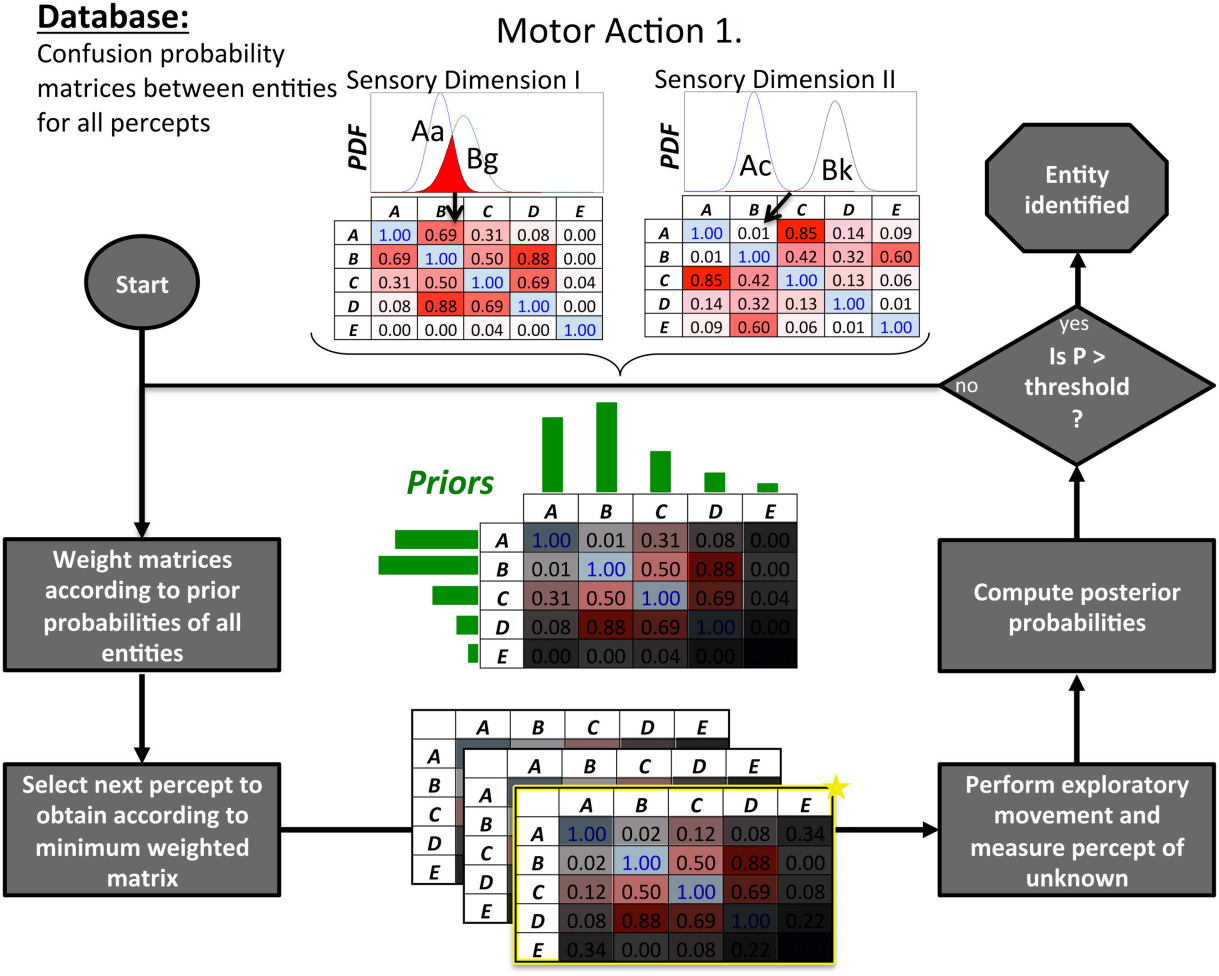
**Bayesian Exploration is used iteratively to identify an unknown entity by selecting and executing exploratory movements to disambiguate alternative hypotheses A–E**. Prior experience with all candidate identities is stored in a database that permits extraction of confusion probability matrices (top center) that quantify the degree of overlap (red) in the probability density functions (PDF) of their sensory percepts (e.g., Aa = sensation a resulting from using motor action 1 with entity A as illustrated in Figure 2). The confusion matrices are then weighted (shaded gray masks) according to the prior probabilities (green bars) of A–E, then summed to identify the percept that will result in the lowest total confusion. Bayesian inference is used to compute the posterior probabilities of A–E to see if one of them exceeds a threshold value; if not, the process is repeated.

**Bayesian Exploration** is an experience-driven algorithm that extends this to decide which next exploratory movement is anticipated to provide the most useful data to disambiguate the current most-probable candidates in an object identification task. It requires a similar experiential database of previously explored objects that associates each object with a set of **percepts**, each percept consisting of one exploratory movement and a particular sensation thereby obtained (see Figure 2). A graphical representation of this algorithm is provided in Figure 3 (full equations in Fishel and Loeb, 2012a). In summary, the degree of perceptual overlap that would result from making each movement and measuring each sensation for each pair of candidate objects is weighted by the current probabilities that the unknown is one of those two candidate objects. The sum of the weighted overlaps for all object pairs reflects the expected residual ambiguity if that percept were to be measured. The percept that is expected to result in the lowest residual ambiguity wins; its action is performed and the corresponding sensation is compared with expectations to update the Bayesian probabilities.

KEY CONCEPT 3. Bayesian Exploration A statistical process derived from Bayesian inference to determine which test is expected to produce evidence that best disambiguates which of several competing hypotheses is true based on the prior probabilities of these hypotheses and a database of experience correlating tests and the resulting evidence for all possible hypotheses (see Figure 3).

KEY CONCEPT 4. Percepts A perceived characteristic of an object resulting from a given test (e.g., exploratory movement) and one aspect of the sensory information that is thereby elicited from that object.

Bayesian probability was first proposed as a mechanism to guide visual exploratory behaviors (Bajcsy, 1988) such as efficiently directing attention to the relevant features of a complex image (Neisser, 1976; Henson et al., 2011; Moreno-Bote et al., 2011) or actively steering the point of view for machine vision (Denzler et al., 2009; Browatzki et al., 2012). Bayesian ideal observer strategy has been applied successfully to explain the patterns of saccadic eye movements that humans use to locate a known, low-visibility object buried in visual noise (Najemnik and Geisler, 2005). Statistically optimal models have been developed to decide what new data to obtain when training a machine learning algorithm (Cohn et al., 1996). Lindley used Shannon information theory to derive a similar approach to deciding which experiment will be more informative (Lindley, 1956).

**Figure 4:**
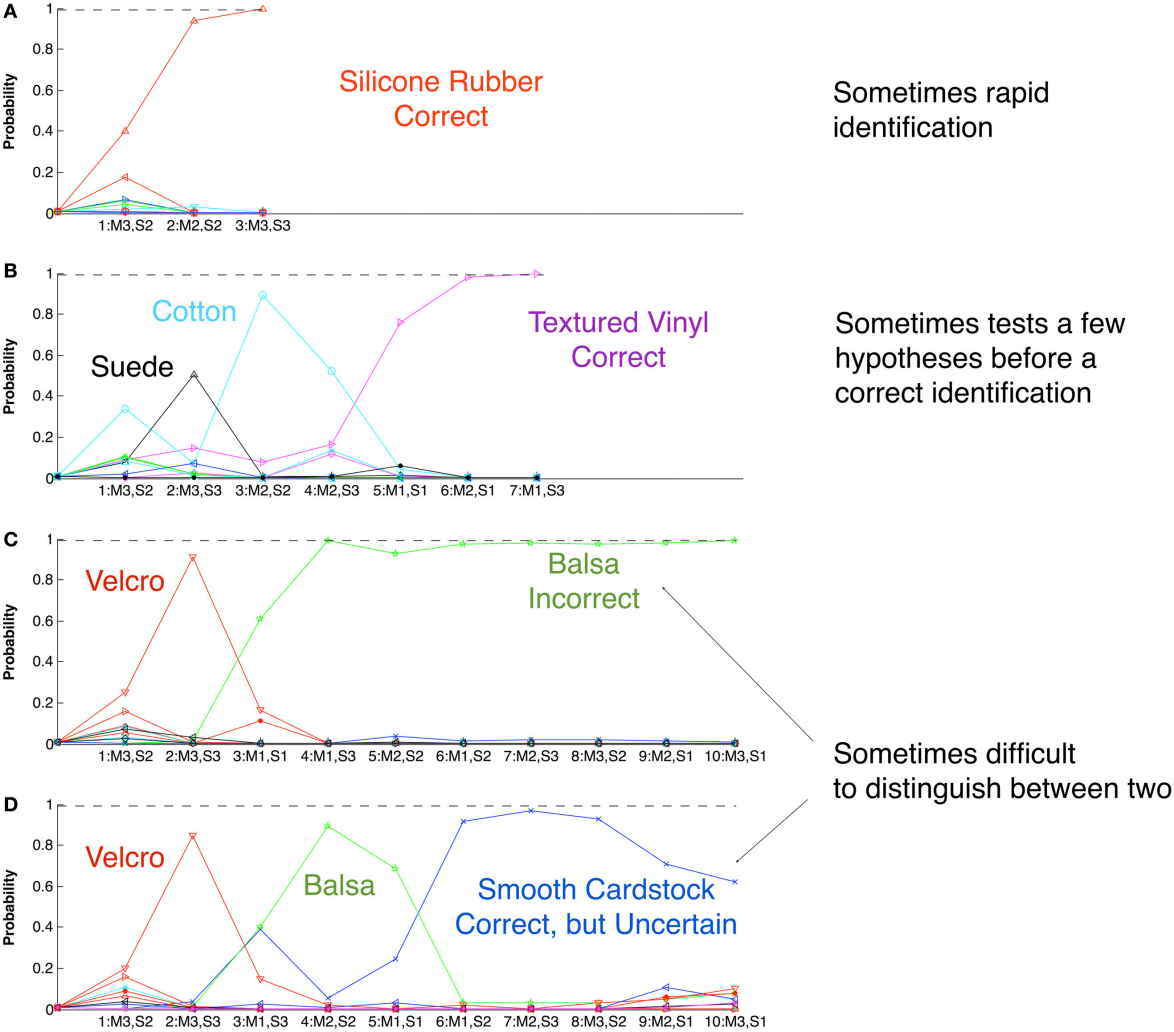
**The performance of the texture identification robot using Bayesian Exploration with a database of 117 textures previously explored with three exploratory movements (M1–3) and characterized according to three sensory percepts (S1–3)**. At the beginning of all four trials **(A–D)** with unknown samples, the probability of any specific texture (ordinate) is 1/117. Each successive movement and sensory percept (numbered pairings along abscissa) is selected according to its potential to disambiguate the currently likely possibilities (see Figure 3), resulting in a new set of probabilities for each remaining viable candidate (colored lines). Similar to humans, the number and sequence of exploratory movements required to make an identification is variable and tends to be influenced by small amounts of noise in the system (trials **A** and **B**). Early in exploration, the particular exploratory movement chosen and percept that is thereby obtained leads to a substantial probability for a material that is then easily rejected by a subsequent movement (suede and cotton in trial **B**; Velcro in trials **C** and **D**). Some materials such as smooth cardstock and balsa wood are not reliably distinguishable by humans or by the robot (trials **C** and **D**). Overall, the robot correctly identified 95.4% of the test materials in a median of 5 exploratory movements.

Bayesian Exploration turned out to be an excellent strategy for our problem of machine touch. It is optimal in the sense that it minimizes the amount of exploration required to reach a given level of confidence in the identity of an unknown but previously experienced entity. It provided a formal computational procedure for the “decision rule” needed to close the perception-action loop (Ernst and Bülthoff, 2004). We built up a robotic experiential database for 117 different texture samples, using a few exploratory movements and sensory features similar to those employed by humans. When unknowns from the sample set were presented, the Bayesian Exploration algorithm resulted in 95% successful identifications, often correctly identifying similar materials that humans could not discriminate despite *ad libitum* exploration. This is better performance on a much larger set than has been reported previously for such machines, which were tested on only 3–20 textures selected to be distinctive and trivial for humans to discriminate (de Boissieu et al., 2009; Giguere and Dudek, 2011; Jamali and Sammut, 2011; Oddo et al., 2011; Sinapov et al., 2011). More importantly for the discussion here, the emergent behavior was qualitatively humanlike. Figure 4 provides examples of indecision, error, and reconsideration similar to human behavior in such tasks (Gallistel et al., 2004; Fishel and Loeb, 2012a). The number of exploratory movements required was small but variable (median ~5), and the sequence changed depending on how the probability estimates were affected by small amounts of noise (Fishel and Loeb, 2012a). Similar variability was observed for optimal visual saccades in a target location model (Najemnik and Geisler, 2005).

Decision-theoretic algorithms based on optimizing Bayesian sequences have been applied to various problems in haptic perception, particularly to cope with ambiguities of machine vision (Schneider et al., 2009; Hsiao et al., 2010; Browatzki et al., 2012) or uncertainties about object location or orientation (Hsiao et al., 2010; Lepora et al., 2013). Tanaka et al. recently took advantage of its ability to learn distinguishing features empirically (Tanaka et al., 2014) rather than requiring *a priori* parameterization (Saal et al., 2010). It can also be applied to autonomous, open-ended classification without requiring supervised learning of a specific database (Dallaire et al., 2014). The Bayesian theoretic approach is agnostic as to sensory modality, so it can be applied easily to multimodal perception (Ernst and Bülthoff, 2004; Sinapov et al., 2014).

## Action for perception vs. perception for action

If perception necessarily involves figuring out what (exploratory) movement to make, then what is action? We submit that they are the same computational process but with different motivation. Consider a caveman walking along a rocky trail and encountering a rabbit that might provide dinner. The caveman will have a very sophisticated classification system for rocks and pebbles of various shapes and textures, but what matters in the moment is to pick up a rock that is suitable for throwing accurately before the rabbit hops away. As the caveman gropes on the ground without looking away from the rabbit, his fingers have fleeting encounters with various objects: twigs, boulders, gravel, rocks. Rather than identifying each object individually, the caveman seeks out the first object that has the requisite **affordances** to complete his goal; this is the perception phase. Most are immediately discarded as unsuitable because they do not afford accurate throwing with sufficient kinetic energy to disable the rabbit, based on previous experience performing this task. The groping is designed specifically to produce a distinctive set of sensory signals from objects that might be suitable. Upon encountering a suitable candidate, the caveman may quickly make another exploratory movement such as hefting to generate sensory data that is specific to the most important property of previously thrown objects. In order to execute the throw, the caveman does not need to compute the requisite motor program by solving the inverse dynamics of the limb now loaded with the rock; instead he executes the previously learned program that is currently in his mind's eye (Loeb, 2012). The caveman moves smoothly from object perception and identification to object manipulation and task performance. Rather than using previous experience to recall the movement that would yield the percepts most likely to identify an object, the look-up process is used instead to recall a movement that will result in the desired sensory feedback when handling the object.

KEY CONCEPT 5. Affordances The association of an object with the various actions and functions that it enables. For example, any object with a certain size, density, and strength affords its use as a hammer because it can be accelerated and collided repeatedly with other objects to convey energy and/or momentum into the other object. It would also afford use as a doorstop.

Psychophysicists and neurophysiologists historically treated action and perception as fundamentally different behaviors under the control of different parts of the nervous system, but the concept of learned “ideomotor” associations (dating from the nineteenth century) has been gaining ground (Melcher et al., 2012). The ideomotor concept—physical actions arising without conscious intent—lends itself well to largely subconscious behaviors such as saccadic eye movements and haptic exploration. More recently psychologists have emphasized the active nature of sensory experience (O'Regan and Noë, 2001; Engel et al., 2013); see Active vs. Passive Sensory Experience below. Bayesian Action&Perception takes Bayesian Exploration one step further to converge “action for perception” with “perception for action.” Bayesian Exploration requires that an **associative memory** be searched to identify the motor program that will generate patterns of sensory feedback that are most distinctive for the probable identities of the object. It seems plausible that the same memory can be searched to find the motor program that will most likely result in a particular pattern of sensory feedback associated with successfully attaining the desired goal once the perceptual ambiguity is removed. Such a capability would be facilitated by preprocessing the sensory representation to reflect the critical control points in the related motor program, as seems to occur when songbirds listen to vocalizations similar to those they make themselves (Amador et al., 2013). Cohn et al. (1996) pointed out that representing learned information as a mixture of Gaussians (similar to the normal distributions used to represent sensory percepts in Bayesian Exploration; see Figure 3) made it possible to “invert” a learned model of input-output relationships by conditioning on the outputs of the model. If the brain can build and search such an associative memory of previously experienced objects, this could provide immediate access to information about the affordances of those objects (Gibson, 1977, 1988; Cisek, 2007), even before the ambiguities of object identity were fully resolved.

KEY CONCEPT 6. Associative memory A biological or electronic mechanism for storing and recalling multidimensional information about entities. For example, previous experience with representatives of a particular entity will consist of concurrences between specific motor programs whereby the representatives have been handled and resulting sensory data. When one of these elements (e.g., the entity identifier, the sensory data or the motor program) is entered into the associative memory as an index, the other elements associated with that index item are recalled as outputs.

The traditional division of sensory, motor, and associational cerebral cortex flies in the face of their obviously common phylogeny and ontogeny and their similarities in cytoarchitecture and external connectivity (Diamond, 1979). Friston and colleagues have been developing a unifying model based on prediction error (Adams et al., 2013), originally introduced to interpret oculomotor behavior in visual scene analysis (Rao and Ballard, 1999). All of our interactions with the world can be expressed as comparisons between the sensory information that we receive and that which we predict based on our internal representation of the cause-and-effect relationships that we think exist. Some of these predictions manifest invisibly as subcortical neural computations while others are accompanied by overt movements, but they are all computationally the same process. The “motor program” that is recalled and regenerated to throw the rock at the rabbit is a manifestation of the prediction that this motor output will produce the expected set of sensory feedback: the feel of the rock leaving the fingers, the visual trajectory and the sound of the impact. This is not fundamentally different from looking upward from the nose of the rabbit to confirm that it has long ears as expected.

## A unifying explanation for mirror neurons

The convergence of perception and action may shed light on the strange proliferation of **mirror neurons** in the nervous system. The name was originally applied to premotor cortical cells that fired similarly when a given movement was made by a monkey or when the monkey observed another person making that movement (di Pellegrino et al., 1992; see review Oztop et al., 2013). However, similar activity occurs also during a hold period between telling the subject what act will be performed and providing a GO command to initiate the act. Both mirror-like and hold-period activity have been observed in virtually all sensory, motor, and association areas of cortex in which researchers have looked (Casile, 2013), as well as in the interneurons of the spinal cord (Prut and Fetz, 1999). Another interpretation is that this activity reflects a motor “SET” command that prepares the system to make the observed or planned movement but falls short of or actively inhibits the actual generation of the movement (Vigneswaran et al., 2013). The exchange of preparatory information with lower motor centers such as the spinal cord may be a necessary part of the dynamics of movement generation (Churchland et al., 2010). It may be required to handle the large number of gains to be set in the spinal interneuronal circuitry in order to achieve behavior that is well-coordinated by sensory feedback (Raphael et al., 2010).

KEY CONCEPT 7. Mirror neurons Neurons that respond to the conjunction of real or imagined motor and sensory behavior. Examples include observing the performance of a task and planning the delayed execution of a task.

Bayesian Action&Perception hypothesizes that thinking about an object in any way inevitably activates all the memories associated with it, which include the dynamic sequences of movements that have been made with the object and the sensory data that resulted from such experiences. Those sensory data are discriminative only in the context of imagining the self-made movements that led to them, so mirror-neuron-like activity should be a part of any recall associated with sensory perception. Conversely, the selection of a specific goal-directed movement is possible only after evaluating the likelihood that it will lead to the sensory feedback associated with attaining that goal (Bonaiuto and Arbib, 2010). The diverse details of mirror neuron activity distinguished by Oztop et al. (2013) would then reflect the computation normally performed by the part of the nervous system in which the mirror neurons reside rather than any specific property of the mirror neurons themselves. What distinguishes them is a requirement for active (but not necessarily conscious; see next section) thought, without requiring overt or even planned motor action (see Active vs. Passive Sensory Experience below).

## What do we imagine when we perceive?

The texture identification robot is an example of the utility of Bayesian Exploration (Fishel and Loeb, 2012a), but it only suggests plausibility for a corresponding biological mechanism. Lacking any compelling proof of Bayesian Action&Perception in the brain, let us indulge in some introspection. Imagine groping in a dark room for objects on a table. Upon first contact with an object, its most probable identity flashes into the mind's eye (we use “mind's eye” figuratively to include all of the sensory modalities that might be relevant to the object). That probability is colored by any prior information about the nature of the room (is it an office or a kitchen?). It leads inexorably to a confirmatory exploratory movement, which results in additional sensory data that may reinforce the mind's eye view or cause it to fall apart in favor of another mind's eye image of a different object that is more consistent with the incoming data. Similarly, imagine yourself watching someone else use a tool in a clumsy manner. Your mind races one step ahead, anticipating the awkward slip and picturing what you would have done instead to avoid it. Neural activity consistent with such “active observation” has been recorded in both dorsal premotor (Cisek and Kalaska, 2004) and primary motor cortices (Tkach et al., 2007) of behaving monkeys.

All this mental thrashing in the absence of any actual requirement for sensorimotor behavior might seem like a huge waste of effort. Like Walter Mitty (Thurber, 1939), we imagine ourselves always “in the moment” whether preparing to perform, teaching someone else to perform, or merely empathizing with a character in a movie or a novel. The most obvious advantage is that this mental activity keeps us prepared to step in and act at a moment's notice, a useful skill for a band of hunters. Anyone who has played doubles in tennis or outfield in baseball has experienced the danger of becoming a passive spectator rather than an active participant. Beyond that, it may simply be impossible for us to form any useful memories about an object without simultaneously representing both the sensory data and the behavioral actions that give rise to those data.

As noted above, much of this highly purposive preparation, exploration, and interpretation can occur largely subconsciously, a phenomenon that was long-recognized (Helmholtz, 1867) but has tended to be ignored by more recent focus on a singular “searchlight of attention” (Crick, 1984). Just as we are often unaware of the logic behind or even the existence of exploratory saccadic eye movements, so are we unaware of much of what we do with our hands. Even the details of the sensory information are usually suppressed in favor of the illusion that we are experiencing what is most probable, a phenomenon that is the source of a vast number of sensory illusions in various modalities including tactile (Hayward, 2008). It is only when the sensory data reach some threshold of dissonance with expectations that the whole set of exploratory movements and sensory percepts rises to the level of awareness (MacKay, 1990).

## Can bayesian algorithms be realized in neural circuitry?

The representation of objects as Bayesian distributions of probabilistic states seems attractive for many reasons. The accumulation of perceptual information in the parietal cortex is consistent with Bayesian decision-making (Beck et al., 2008). Its hypothetical realization in associative neural networks and the use of those networks to provide corrective feedback during motor behaviors has been described (Knill and Pouget, 2004).

Let us suppose that the internal representation of each object is the complete associative memory of all motor programs and consequent sensory data associated with the object (Pastor et al., 2011). The size and organization of this associative memory must be considered carefully for this theory to be viable over the huge range of entities and experiences that we accumulate over a lifetime. It seems likely that the descriptions of both the motor behaviors and the sensory data will need to be highly abstracted in some efficient, hierarchical representation such as we might expect from the proliferation of distinct cortical areas in primates.

The number of entities that humans learn to discriminate and the repertoire of movements and percepts that they employ are much larger than those that have been modeled to date. Without careful structuring of the neural representations, this leads to the **curse of dimensionality** (Ganguli and Sompolinsky, 2012). This is the reason that the percepts illustrated in Figure 2 each consist of an individually testable pairing of a single action with a simple sensation rather than simultaneously considering all the sensations that might make up a “gestalt” representation. Representation of learned information as mixtures of Gaussians lends itself to both efficient storage and efficient incorporation of new experiences (Cohn et al., 1996). An effective way for neurons to store sensory data is simply to reinforce the synaptic projections among them that fire in specific temporal relationships (Hebb, 1949). Restricted Boltzmann machines can self-organize complex data sets into hierarchical neural networks that support efficient perception (Hinton, 2012). They can be used to learn associations among multiple sensory modalities (Sinapov et al., 2014) and to account optimally (in Bayesian terms) for differences in their reliability (Makin et al., 2013). Hebbian learning added to a recursive sensorimotor planner results in behaviors that change based on the most recent tasks requested and performed (Verstynen and Sabes, 2011). An efficient way to interpolate many motor behaviors from a sparsely distributed repertoire has been modeled recently (Tsianos et al., 2014), provided that these abstracted representations of motor behaviors can be executed by a well-designed lower sensorimotor system that includes contingency plans in the form of complex reflexes (see below and Loeb, 2012). Marques et al. recently demonstrated that motor programs that include such reflexes can arise from spontaneous motor activity (Marques et al., 2014) such as the random exploratory strategy suggested below. Interpolable motor programs are also consistent with observations that motor behaviors are well-represented by recurring patterns of muscle synergy (Alessandro et al., 2013).

KEY CONCEPT 8. Curse of dimensionality As the number of descriptors (dimensions) associated with an entity increases, the amount of experience required to detect those associations reliably will tend to increase exponentially. This problem is usually addressed by either reducing the dimensionality of the data (which may be computationally expensive) or increasing the amount of training (which may be impractical).

Bayesian Action&Perception requires a mechanism to perform the probability weighted calculation of the most useful next exploratory movement to make. The cerebral cortex isn't likely to be solving the equations behind the machine version of the process illustrated in Figure 3 (Fishel and Loeb, 2012a). In fact, deciding among competing demands for action is a common function performed even for more primitive behaviors. For example, the superior colliculus (midbrain tectum) decides on which saccade to make in the face of multiple salient stimuli in different parts of the visual space (Fecteau and Munoz, 2006). This requires only a highly parallel array of reciprocally inhibitory neurons, a common feature of many parts of the nervous system including the cerebral cortex (Isaacson and Scanziani, 2011). A similar process may be apparent in motor cortical activity as a monkey simultaneously considers multiple trajectories through an obstructed space and finally makes a selection (Churchland et al., 2010).

It is important to distinguish the general strategy of Bayesian decision-making from the tactics of optimality that have been developed to implement it using mathematical algorithms (Loeb, 2012). Optimality is a methodology that rests on a belief system. It requires that our corner of the universe be sufficiently deterministic that there exists an optimal path through it and that the rewards of following that unique path exceed the costs of finding it. Often neither is true. A more appropriate question for this section would have been “Can Bayesian algorithms *be approximated by* neural circuitry?” Interestingly, approximate solutions to computationally intense Bayesian equations are an active research topic in machine learning and robotics (Kwisthout et al., 2011; Bitzer et al., 2014).

### Valuation of action and perception

**Figure 5:**
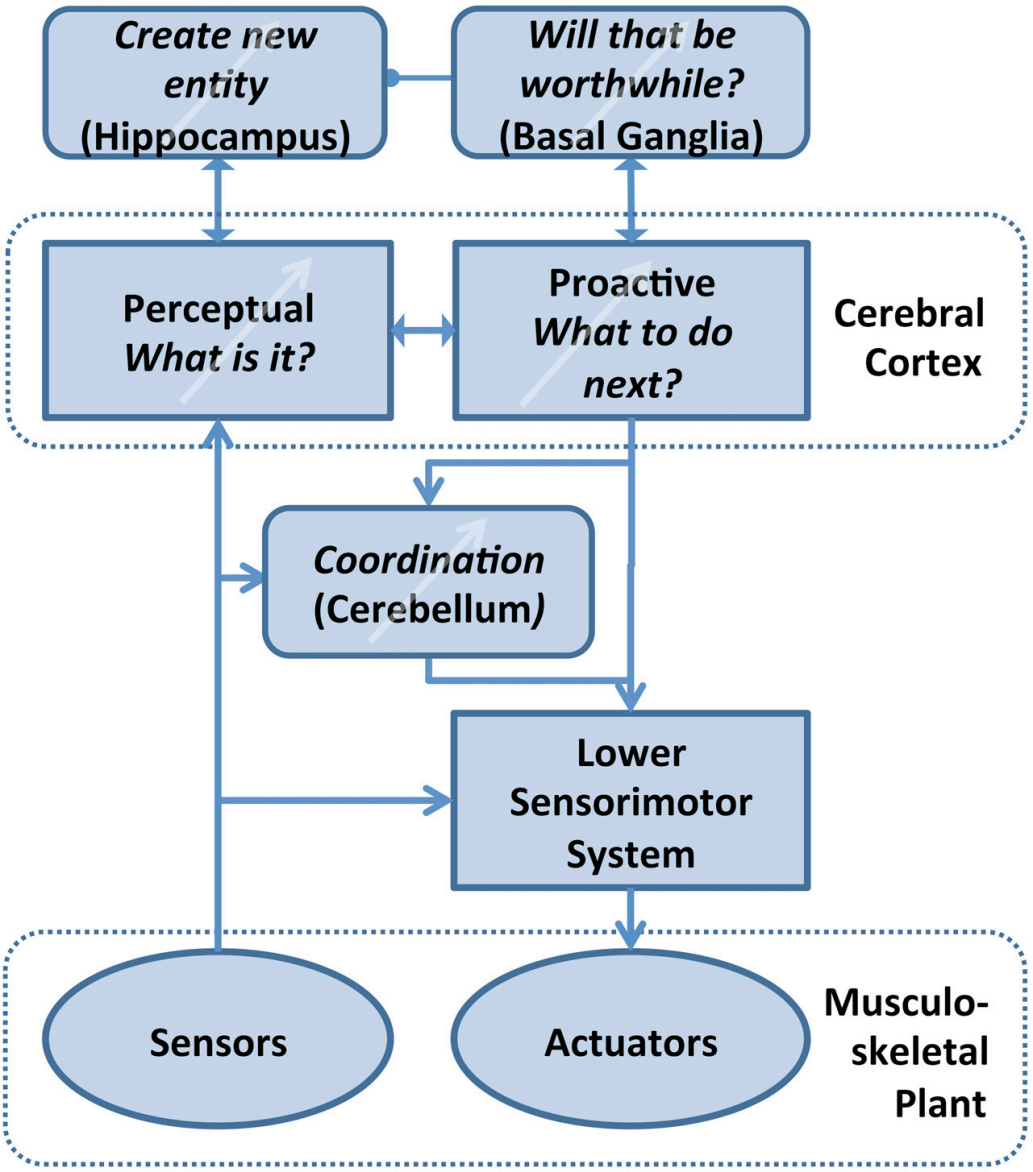
**Theory of computation for Bayesian Action&Perception**. The associative memory in the various areas of cerebral cortex interprets incoming sensory data in the light of current hypotheses about the potential identity of objects (Perceptual) and selects an output that it expects will confirm that hypothesis by generating new sensory data (Proactive). Orderly development, use and refinement of this cortical database requires three major supporting functions that also require some form of learning: Value judgments are required to decide what level of certainty is acceptable for the identity and expected behavior of an unknown object, tentatively ascribed to the basal ganglia (Bornstein and Daw, 2011). If no acceptable identification is possible, then these unreconciled associations of motor strategies and sensory feedback that have been experienced with the unknown object must be remembered and eventually turned into a compressed, efficient representation of a new entity in cerebral cortex, tentatively ascribed to hippocampus (Winocur et al., 2010; Petrantonakis and Poirazi, 2014). The learned, abstract motor strategies need to be coordinated with lower level sensorimotor systems (e.g., spinal cord and brainstem) that can activate and stabilize complex body movements, tentatively ascribed to cerebellum (Thach, 2014).

The confidence level for our texture discrimination task was set arbitrarily to 99%, but an acceptable level would normally depend on motivation, e.g., the trade-off between the need for a speedy decision vs. the consequences of a mistaken identification (see example of throwing a stone above). If an object is sufficiently dissimilar to any previously experienced, the confidence threshold will never be exceeded. At some point, the organism must decide to create in its database a new entity consisting of the percepts (i.e., motor commands and sensory feedback) associated with exploring that entity. This requires some way to save the motor and sensory data associated with those unresolved explorations, as opposed to simply reinforcing the associative memory with the uninteresting observations of familiar objects. Valuation against prior experience and acquisition of new memories are functions that predate the evolution of cerebral cortex and that require global access to multimodal sensory and motor information. This suggests that these supportive functions are performed by subcortical structures; one such scheme is described in Figure 5 and its legend.

At any time, the Bayesian Exploration algorithm must have a current best guess as to what entity is actually present based on whatever priors it has. The confidence threshold merely tells the machine when to stop exploring. Any new physical entity will first be classified as the most likely one of the previously experienced physical entities because those are the only mental entities that the brain has. The brain may then realize that the probability has become stuck below the current confidence threshold. This would imply that a new mental entity should be created within the region of hyperspace currently occupied by the closest extant mental entity. Therefore, the decision to create a new mental entity is essentially the decision to split an extant mental entity into two. The nature of the split and the two new mental entities should not be taken lightly because they now become the basis for the entire future performance and development of the organism. If the split is motivated by a failure to predict an affordance correctly (as proposed above), then the nature and consequences of the failure can be used to provide valuations in order to inform this splitting process. Valuation and enablement have been proposed for the relationship between the motor cortex and the basal ganglia (Doya and Kimura, 2009). Bayesian Action&Perception generates a parallel requirement for sensory cortical regions, which are known to have similar connectivity with basal ganglia (Bornstein and Daw, 2011).

The texture discrimination robot described by Fishel and Loeb (2012a) starts with a complete and static relational database, but a real brain must acquire such a database from scratch and must continuously update it to deal with newly experienced objects and changing performance requirements. At an early developmental stage when experience has been limited and the internal representation of the world is very sparse, there will be a natural tendency to accept quickly that a new physical entity is one of the few, already experienced entities that are represented by a corresponding mental entity. If the new physical entity does not then behave as predicted by the mental entity, there will also be a natural tendency to create quickly a new mental entity in the database. As the database fills out with more experience, more exploration will be required to discriminate among similar physical entities and there will be less motivation to create new mental entities. It is tempting to ascribe age-specific tendencies such as “jumping to conclusions” and “being stuck in a rut” to the predictable ontological consequences of Bayesian Action&Perception.

### Iteration rate

The rate at which successive exploratory movements are executed has interesting parallels with temporal phenomena observed in cerebral cortex. Cortically mediated, exploratory saccadic eye-movements tend to occur about 3 times per second, whereas subcortically mediated express saccades can occur in 100 ms (Munoz and Wurtz, 1992). The number of scene details that can be discovered and recalled at the cortical saccade rate is quite low (Melcher and Kowler, 2001), suggesting that the brain is already looking for something specific rather than just collecting information. The identification of significant or “oddball” items (i.e., something that agrees or disagrees with a strong expectation) in a complex image is associated with the P300 EEG wave, a positive potential occurring over the relevant cortical area 300 ms after the stimulus is presented (Chapman and Bragdon, 1964). Both of these cortical phenomena are automatic and subconscious in their details but enabled by mental concentration. It is true that cortical systems are capable of pipelining information to achieve higher rates of throughput such as when processing tachistoscopic images at 8–10 frames/s (Farwell and Donchin, 1988). Subcortical and even cortical loops are capable of generating previously programmed reflexive and corrective responses at 60–100 ms latency (Gribble et al., 2002; Scott, 2004; Johansson and Flanagan, 2007; Kurtzer et al., 2008; Perfiliev et al., 2010). Iterative decision-making such as Bayesian Exploration, however, seems likely to be limited to ~3 sensorimotor cycles/s. This would allow time for physical exploration plus acquisition of sufficient data from all sensory modalities and processing by their respective cortical areas to contribute relevant information.

### Active vs. passive sensory experience

The effectiveness of active vs. passive touch has been long debated. While the importance—even the inevitability—of integrating self-generated movement with tactile sensing and perception has long been recognized (Katz, 1925; Jones and Lederman, 2006; Prescott et al., 2011), humans can perform passive tactile discrimination tasks quite well as long as the applied movement is predictable and relevant to the perceptual discrimination task, as is often constructed for psychophysical experiments (Lederman, 1981). This capability is not surprising because “passive touch” is really a degenerate case of active tactile discrimination. If the object itself is moving, then the subject's exploratory movement is simply to not move. As long as the relative motion between the object and the fingertip is relevant and similar to what a human would select to perform on a stationary object, such discrimination performance is unsurprising. Passive touch performance might even be superior if the movements controlled by the apparatus have less motor noise and variability than would otherwise be produced by active touch (Fishel and Loeb, 2012a). However, passive touch breaks down when the movement of the object does not yield relevant information for the discrimination and the observer is powerless to select another exploratory movement that may gain information.

Theories of visual information processing have become progressively more complex as they contend with changes in the apparent receptive fields of cortical units as a result of attention with and without overt eye movements (Reynolds and Heeger, 2009). Rather than interpreting these effects as a modulation of the visual response, perhaps the data can instead be interpreted as visual feedback modulating an expectation that simultaneously motivates an exploratory behavior. That exploratory behavior might be manifest as actual saccadic eye movements, but only when not suppressed by the experimental design. It would also be accompanied by a “mind's eye” visualization of the expected stimulus (Adams et al., 2013), not unlike mirror neuron behavior. The actual visual input would then be compared to the expected stimulus to confirm or refute the current mental hypothesis about the source of the stimuli (Adams et al., 2013).

Similarly to vision, tactile afferent activity appears to be processed differently by somatosensory cortex depending on whether it arose from passive or active touch (Chapman, 1994; Ackerley et al., 2012). If active exploratory movements are a manifestation of attention in the tactile domain, then this contingent behavior of somatosensory cortex may reflect the same mental hypothesizing as in visual cortex. The locus of that hypothesis formation is likely in other, “associational” cortical areas. Attention-based visuomotor planning has been attributed to frontal eye fields (Zhou and Desimone, 2011). If there were a corresponding haptic planning area, a lesion there might be expected to produce a pure tactile apraxia. Such an apraxia was described in a stroke patient who had essentially normal tactile perception and visual shape comprehension and intact voluntary hand movements but could not describe the shape of a hidden 3D object that she was exploring manually (Valenza et al., 2001). It would appear that haptic knowledge of the world requires more than the sum of the observable parts.

## What types of entities can be represented?

Is it too much of a stretch to expand the Bayesian Action&Perception scheme from physical objects that can be manipulated to all entities that the brain has learned to identify in its world? The human mind is particularly adept at understanding both the physical and the interpersonal world at highly abstracted levels. In order to reach such levels of abstraction, it seems necessary for the brain to hypothesize that certain abstractions exist and then to observe that there exists a class of apparently dissimilar situations that nonetheless lead to similar sensory feedback when handled in a certain way. For example, if one wants to identify whether a newly encountered person is friend or foe, it is probably useful to make a neutral gesture like smiling or extending an open hand to see what response is thereby elicited. Bayesian Exploration lends itself well to integrating multimodal sensory data (Xu et al., 2013; Sinapov et al., 2014), so it might serve as well to integrate multiple levels of abstract perception.

Bayesian decision-making has been criticized as a theory of artificial general intelligence (also known as “strong” AI) because it is fundamentally limited to induction and extrapolation rather than creative perception (Deutsch, 2012). We propose (but have not yet formally demonstrated) that the inverse processes of Bayesian Exploration and Bayesian Action&Perception can overcome this limitation. Consider a hierarchical system in which various ways of exploring an object are themselves entities selected from initially random behaviors (Marques et al., 2014) according to how efficiently they characterize a class of objects (in contrast to the arbitrary exploratory movements that we programmed into our texture identification machine). The percepts that are thereby selected will then depend on the distribution of properties in the particular set of objects in the machine's universe rather than *a priori* knowledge. That is to say that the machine has discovered an abstraction about the design rather than just the contents of its universe.

## What can robotics contribute to neuroscience?

The opening question (“Can robots perceive *in the same manner* as humans?”) implies not just equivalence of performance but also of means. If the function being studied is sufficiently sophisticated and the test is sufficiently rigorous, then perhaps the performance equivalence proposed for the Turing test of humanlike artificial intelligence (Turing, 1950) can only be achieved through closely related means (but see Deutsch, 2012 for fundamental limits of such a demonstration and Horsman et al., 2014 for an alternative approach to experimental testing of theories of computation). While the computational machinery of robots is obviously different from that of neurons, there is no reason why they couldn't use the same theory of computation (Marr, 1982) to structure the problem in the first place.

Haptic functions are difficult to study using the usual reductionist recording methods in trained subjects (Loeb et al., 2011). In addition to the usual problems of measuring both the neural activity and the kinematic details of these complex behaviors, it seems likely that the neural activity will depend on internal states that cannot be controlled or measured. These include the entire prior experience of the subject and the internal hypotheses that are being tested at any point in time. An alternative experimental strategy is to test theories of computation for such mental functions by building biomimetic machines (i.e., autonomous robots) and by observing whether they generate biosimilar behaviors (Avraham et al., 2012; Fishel and Loeb, 2012a; Su et al., 2012). At the least, this should demonstrate the feasibility, if not the existence, of the hypothesized neural algorithms. As a bonus, it might result in some particularly useful machines.

### Conflict of interest statement

Both authors are equity partners in SynTouch, LLC, which manufactures and sells the BioTac sensors mentioned in this article.
